# Acupuncture and moxibustion for pain relief and quality of life improvement in patients with knee osteoarthritis: A protocol for systematic review and meta-analysis

**DOI:** 10.1097/MD.0000000000020171

**Published:** 2020-05-29

**Authors:** Shirui Cheng, Jun Zhou, Guixing Xu, Ming Xin, Ying Cheng, Yuzhu Qu, Yuanfang Zhou, Mi Liu, Xiaorong Chang, Mailan Liu

**Affiliations:** a The Acupuncture and Tuina School, The 3rd Teaching Hospital, Chengdu University of Traditional Chinese Medicine; b The Rehabilitation Department, Chengdu Fifth People's Hospital, Chengdu, Sichuan, China; c The Cheng Clinic Limited, London, England; d The First Affiliated Hospital of Chengdu University of Traditional Chinese Medicine, Chengdu, Sichuan, China; e The College of Acupuncture & Moxibustion and Tuina, Hunan University of Chinese Medicine.

**Keywords:** acupuncture, knee osteoarthritis, meta-analysis, moxibustion, pain relief, quality of life, systematic review

## Abstract

**Objective:**

The aim of this systematic review and meta-analysis is to assess the efficacy of acupuncture and moxibustion in pain relief and quality of life improvement of knee osteoarthritis (KOA) patients.

**Methods:**

The following databases will be searched: MEDLINE, EMBASE, the Cochrane Library, Web of Science, Chinese Biomedical Literature Database, Chinese Nation Knowledge Infrastructure, Wanfang Database, the Chongqing VIP from inception to May 1, 2020. All randomized controlled trials (RCTs) used acupuncture or moxibustion to relieve pain and improve quality of life (QoL) among KOA patients will be included. Study selection, data extraction, quality assessment and assessment of risk bias will be performed by 2 reviewers independently. Data synthesis will be performed using Review Manager V5.3 software. A meta-analysis will be performed when there is sufficient available data.

**PROSPERO registration number:**

CRD42020169724

## Abstract 2

Supplemental Digital Content is available in the text

## 1 Introduction

Knee osteoarthritis (KOA) is a common form of osteoarthritis, often caused by focal loss of articular cartilage, marginal, and central new bone formation.^[1,2]^ Most patients with KOA are associated with pain, swelling, dysfunction, and deformity in knee joint clinically.^[2]^ The global age-standardized prevalence of KOA is 3.8%,^[3]^ affecting more than 9 million individuals in the United States.^[4]^ Pain is the main symptom of KOA and is the first reason for patients to go to their doctors, which affects the quality of life of patients seriously and might induce physical disability of knee furtherly.^[1,5]^

The main interventions to treat KOA including patient education, non-pharmacological therapy, pharmacological therapy, and surgery.^[1,6–8]^ The widely-used pharmacological treatments, non-steroidal anti-inflammatory drugs (NSAIDs), are unsatisfactory with their significant side effects.^[9]^ Although knee replacement is effective and successful in most cases,^[10]^ 20% of patients remain unsatisfied at 1 year after a technically successful procedure.^[11]^

Hence, non-pharmacological approaches are more and more popular to be used to manage chronic knee pain typically due to knee osteoarthritis.^[12,13]^ Acupuncture and moxibustion, the most common complementary and alternative therapy, have significant effects on KOA, including pain relief,^[14,15]^ periarticular swelling reduction,^[16]^ knee function,^[15]^ and quality of life (QoL) improvement.^[17]^ However, the efficacy of acupuncture and moxibustion exist inconsistency. A study published in JAMA in 2014 has reported that acupuncture conferred no benefit over sham for pain or knee function.^[18]^ Several reviews of acupuncture and moxibustion for chronic knee pain have been published from 2007 to 2017.^[19,20]^ The systematic review in 2017 concluded that acupuncture may be effective for chronic knee pain, but it was unable to draw any strong conclusions because of heterogeneity and methodological limitations.^[20]^

In this study, we will perform a systematic review and meta-analysis of double-blind, randomized, controlled studies of acupuncture, and moxibustion that presented data on pain relief and quality of life improvement among knee osteoarthritis. We will compare the differences in pain relief and quality of life improvement in KOA patients who received acupuncture and/or moxibustion treatments with those received sham acupuncture or moxibustion, placebo control, or other active therapies.

## 2 Methods and design

### 2.1 Inclusion criteria

#### 2.1.1 Type of studies

Randomized controlled trials (RCT) reported in English or Chinese will be included. Other study design will be excluded. There will be no restriction on publication date.

#### 2.1.2 Type of participants

Studies enrolling adult patients (aged ≥ 38 years) diagnosed as knee osteoarthritis will be included. There will be no restrictions on age, gender, race, or nation.

#### 2.1.3 Type of interventions

The acupuncture and moxibustion of the experimental group must include forms of needle insertion including fire needle, floating needle, electroacupuncture, etc. or moxibustion at acupoints or trigger points. Besides, acupuncture and moxibustion plus other interventions will also be included. There will be no restrictions on the retaining time, frequency, sessions of acupuncture, and moxibustion.

#### 2.1.4 Type of comparators

The control group with sham acupuncture or moxibustion, placebo control, or other active therapies will be included. There will be no restrictions on the retaining time, frequency, sessions of comparators.

#### 2.1.5 Outcome measurements

Primary outcomes will include the changes in the Visual Analogue Scale (VAS) scores, the McGill Pain Questionnaire (MPQ) scores and the changes in the Short Form health survey (SF-36) scores from baseline to the available follow-up. Secondary outcomes will contain the changes in the Western Ontario and McMaster Universities Osteoarthritis Index (WOMAC) and the incidence rate of adverse events from baseline to the available follow-up.

### 2.2 Exclusion criteria

Non-RCTs, quasi-RCTs, crossover trials, case report, animal studies, experts experience, conference articles will be excluded. Studies comparing the effect of different acupuncture or moxibustion therapies will be excluded. Studies comparing the effect of acupuncture and moxibustion will also be excluded. Studies with a sample size of less than 10 subjects in each group will be excluded.

### 2.3 Search strategy

#### 2.3.1 Electronic searches

From the inception to May 1, 2020, the following databases will be searched: MEDLINE, EMBASE, the Cochrane Library, Web of Science, Chinese Biomedical Medical Database, Chinese Nation Knowledge Infrastructure, Wanfang Database, the Chongqing VIP. The searching strategy of MEDLINE is presented in Supplemental Digital Content (Appendix 1, http://links.lww.com/MD/E229). This search strategy will be modified to be suitable for other electronic databases.

#### 2.3.2 Searching other resources

Unpublished or ongoing trial data will also be searched from the following clinical trial registries: The National Institutes of Health clinical registry Clinical Trials, the Chinese clinical registry, the Australian New Zealand Clinical Trials Registry, the International Clinical Trials Registry Platform. Ambiguous literatures and reference lists of identified publications will be checked manually.

### 2.4 Data collection

#### 2.4.1 Studies selection

**Figure 1:**
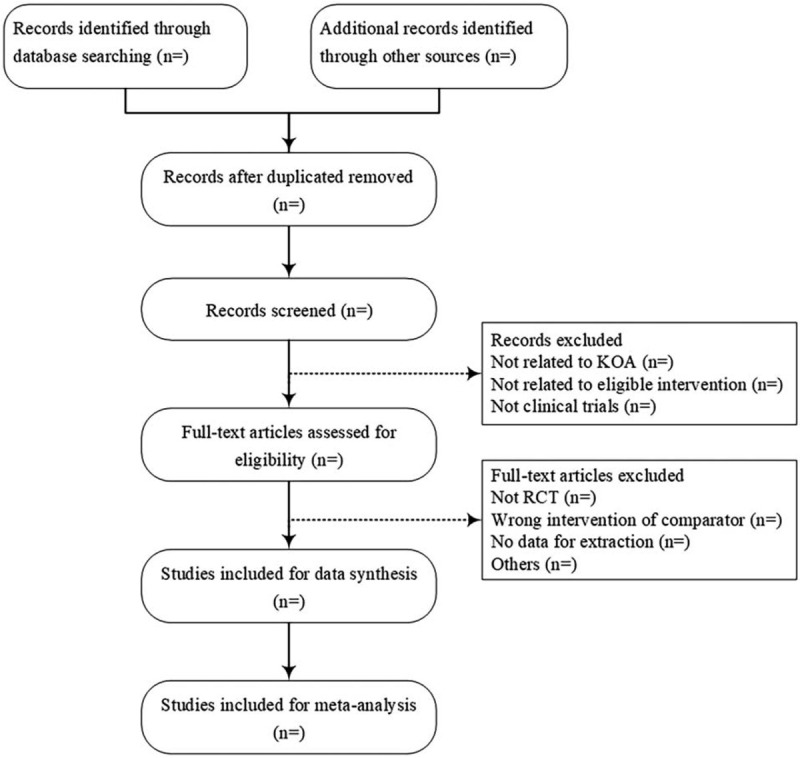
Flow diagram of study selection process.

All the studies of electronic searches and other sources will be imported to Endnote version X9 software, and the duplicated studies will be filtered. The titles and abstracts of potentially qualified studies will be screened by two reviewers (SC and JZ) independently. And the studies not meeting the inclusion criteria will be excluded. The full text will be further screened if the studies cannot be estimated according to the titles and abstracts. After screening, two reviewers will cross-check the included studies. The inconsistent opinions between the two reviewers will be resolved through discussion or a third reviewer (GX). Details of the selection procedure for studies are shown in a Preferred Reporting Item for Systematic review and Meta-analysis protocol (PRISMA-P) flow chart (Fig. 1).

#### 2.4.2 Data extraction

The following information from the included studies will be extracted by two reviewers (SC and MX) using a standard form: the first author, year of publication, country, funding supports, the mean age of participants, sex, ethnicity, education, illness duration, study design, sample size, diagnostic criteria, method of randomization, blinding, interventions and controls, method of analysis, outcome measures, mean and SD of outcomes, adverse events. The disagreement between the two reviewers will be solved by discussion or a third reviewer (GX). The extraction data will be listed in Microsoft Excel, and JZ will check the entered data to ensure the consistency and correct data entry errors.

### 2.5 Risk of bias assessment

The risk of bias of the included trials will be evaluated by two reviewers (SC and GX) independently using the Cochrane risk of bias tool (http://www.cochrane-handbook.org). The following 6 items will be assessed, including: random sequence generation, allocation concealment, blind subjects and therapists, blind assessors, incomplete outcome data, selective reporting, and other bias when required.^[21]^ The risk of bias in each item is rated as “high”, “low risk”, or “unclear of bias”. The disagreements will be resolved through discussion or the third reviewer (MX).

### 2.6 Dealing with missing data

If the data of the primary studies is missing, the authors will be contact for the information. If the missing data cannot be obtained, the studies will only be included for narrative analysis.

### 2.7 Quality of evidence assessment

The quality of evidence of outcomes will be assessed by two reviewers independently according to the Grading of Recommendations Assessment, Development and Evaluation (GRADE) system. The GRADE system includes 5 items: the risk of bias, inconsistency, indirectness, imprecision, and publication bias.^[22,23]^ The quality of evidence will be rated as “high”, “moderate”, “low” or “very low”.

### 2.8 Data analysis

#### 2.8.1 Data synthesis

To summarize the effects of acupuncture and moxibustion on outcomes, the weighted mean differences (WMD) with 95% confidence interval (CI) from each study will be synthesized using Review Manager version 5.3 (RevMan V5.3, the Nordic Cochrane Centre, Copenhagen, Denmark). The relative risk (RR) with 95% CI will also be calculated. The variance of the change will be imputed using a correlation factor of 0.4 as suggested by the Cochrane Collaboration. If excessive statistical heterogeneity is not existing, we will pool data across studies using fixed-effects model for meta-analysis. When statistical heterogeneity exists, a random-effects model will be used for meta-analysis. Besides, a narrative and qualitative summary will also be provided.

#### 2.8.2 Assessment of heterogeneity

The heterogeneity will be analyzed through Chi-Squared (*X*^2^) test by using RevMan V5.3 according to the Cochrane Handbook. *P* value of less than 0.10 will be considered significant. Moreover, we will calculate the *I*^2^ value on the meta-analysis to quantify the impact of the statistical heterogeneity. If the *I*^2^ value is over than 40%, the potential cause of the significant heterogeneity will be further explored using meta-regression analysis.

#### 2.8.3 Subgroup analysis

If the number of included studies is enough, subgroup analysis will be conducted according to the different types of acupuncture and moxibustion, age, sex, control interventions, treatment frequency.

#### 2.8.4 Sensitivity analysis

In order to verify the stability of the primary outcomes, sensitivity analysis will be performed according to the sample size, study design, methodological quality, the effect of missing data of the included studies.

#### 2.8.5 Assessment of publication biases

Funnel plots will be performed to assess the reporting bias when more than 10 trials are included. If the funnel plots are asymmetric, we will try to interpret the funnel plots asymmetry.

## 3 Discussion

KOA is a common chronic degenerative disease of knee joint in elderly,^[24]^ which is significantly impairs the physical function and QoL of target population.^[25,26]^ Current regimes are aiming to reliving symptom and recovering physical function because KOA cannot be cured.^[10,27]^ Previous reviews have been performed to compare the effectiveness and safety between moxibustion and sham control and conducted that moxibustion treatment is equal to the oral drugs and intra-articular injections in treating patients with KOA.^[28,29]^ Another updated systematic review has assessed the effectiveness and safety of acupuncture for the treatment of chronic knee pain.^[20]^ Chronic knee pain can be induced due to knee osteoarthritis, rheumatoid arthritis, urarthritis, meniscus injury, etc., which may bring about the heterogeneity limitations of the included trials in the above review.^[20]^

Hence, this study aims to investigate effectiveness of acupuncture and moxibustion for relieving pain and improving the QoL of the patients with KOA. The results of this SR will provide more reliable evidence of acupuncture and moxibustion for relieving pain and improving the QoL, in order to better understand the potential benefits of acupuncture and moxibustion and broaden the clinical application. The results of this review will be reported according to the Consolidation of Standard for Reporting Trials guidelines (CONSORT)^[30]^ and recommendations described in PRISMA statement and the Cochrane Handbook for Intervention Reviews.

There are some limitations in this review. Different types of acupuncture and moxibustion may cause considerable heterogeneity in this systematic review. So subgroup analysis will be performed based on the type of acupuncture and moxibustion if possible. Because of the barrier of language, only trials published in English or Chinese will be included.

## Author contributions

**Conceptualization:** S Cheng, M Liu.

**Data curation:** S Cheng, J Zhou, G Xu, M Liu.

**Methodology:** S Cheng, J Zhou, G Xu, M Xin, M Liu.

**Project administration:** G Xu, Y Cheng.

**Writing – original draft:** S Cheng, J Zhou, G Xu.

**Writing – review & editing:** M Xin, Y Qu, Y Zhou, Mi Liu, X Chang, M Liu.

## Supplementary Material

SUPPLEMENTARY MATERIAL
