# Supplementary material for: Acupuncture and moxibustion for pain relief and quality of life improvement in patients with knee osteoarthritis: A protocol for systematic review and meta-analysis
Source: Medicine (Baltimore). 2020 May 29;99(22):e20171. doi: 10.1097/MD.0000000000020171 (PMC12245213; doi:10.1097/MD.0000000000020171)
Supplement: SUPPLEMENTARY MATERIAL [file medi-99-e20171-s001.docx]

**MEDLINE search strategy**

1. acupuncture therapy/ or acupressure/ or acupuncture analgesia/ or acupuncture, ear/ or electroacupuncture/ or meridians/ or moxibustion/
2. Acupuncture/
3. acupuncture.tw.
4. (electroacupuncture or electro-acupuncture).tw.
5. acupoint$.tw.
6. ((meridian or non‐meridian or trigger) adj5 point$).tw.
7. needl$.tw.
8. moxbustion.mp. or *Moxibustion/
9. *Moxibustion/ or moxa.mp
10. or/1‐9
11. exp osteoarthritis/
12. (osteoarthriti$ or osteoarthro$ or gonarthriti$ or onarthro$ or coxarthriti$ or coxarthro$).ti,ab,sh.
13. (arthros$ or arthrot$).ti,ab.
14. ((knee$ or hip$ or joint$) adj3 (pain$ or ach$ or discomfort$)).ti,ab.
15. ((knee$ or hip$ or joint$) adj3 stiff$).ti,ab.
16. or/11-15
17. randomized controlled trial.pt.
18. controlled clinical trial.pt.
19. randomized.ab.
20. placebo.ab.
21. drug therapy.fs.
22. randomly.ab.
23. trial.ab.
24. groups.ab.
25. or/17‐24
26. (animals not (animals and humans)).sh.
27. 25 not 26
28. 10 and 16 and 27
